# circKIF4A sponges miR-127 to promote ovarian cancer progression

**DOI:** 10.18632/aging.103389

**Published:** 2020-08-05

**Authors:** Shunliang Sheng, Yi Hu, Furong Yu, Wenjuan Tong, Shufen Wang, Yanlin Cai, Jiayu Zhu

**Affiliations:** 1 Department of Gynaecology and Obstetrics, The First Affiliated Hospital of University of South China, Hengyang, Hunan, China; 2 Department of Obstetrics and Gynecology, Nanfang Hospital of Southern Medical University, Guangzhou, Guangdong, China

**Keywords:** ovarian cancer, circKIF4A, ceRNA, miR-127, JAM3

## Abstract

Ovarian cancer is a major gynecologic cancer and common cause of gynecologic cancer death worldwide. However, the molecular mechanisms of ovarian cancer progression are still unclear. circular RNAs (circRNAs) are recently reported to be involved in cancer progression regulation but the potential functions of circRNAs in ovarian cancer remains unknown. In this study, we explored the expression of circKIF4A in ovarian cancer tissues. Then, a series of experiments were conducted to investigate how circKIF4A functioned in ovarian cancer *in vitro* and *in vivo*. The results revealed that circKIF4A was highly expressed in ovarian cancer tissues. Knockdown of circKIF4A suppressed cell proliferation and migration in ovarian cancer. Subsequent mechanism study revealed that circKIF4A acted as a competitive endogenous RNA (ceRNA) to promoted ovarian cancer progression by sponging miR-127 and upregulated the expression of Junctional adhesion molecule 3 (JAM3). Therefore, circKIF4A could be a novel biomarker and therapeutic target for ovarian cancer.

## INTRODUCTION

Ovarian cancer is one of the major gynecologic cancers and common cause of gynecologic cancer death worldwide [1]. In 2018, there are 295,414 new cases and 184,799 death of ovarian cancer around the world [2]. To date, the prognosis of ovarian cancer is still poor. Exploring the molecular mechanisms of ovarian cancer progression and developing novel strategies are needed.

Recently, it has been reported that circular RNAs (circRNAs) are involved in cancer progression regulation [3], such as in breast cancer [4] and in prostate cancer [5]. circRNAs could also act as biomarkers to help cancer diagnosis and predict prognosis [6]. However, the potential functions of circRNAs in ovarian cancer remains unknown. Moreover, plenty of studies have indicated that circRNAs regulate gene expression by sponging microRNAs and act as competitive endogenous RNAs (ceRNAs) [7]. In triple-negative breast cancer, circKIF4A acts as a ceRNA for KIF4A via sponging miR-375 to promote cancer progression [8]. But how does circKIF4A function in ovarian cancer is still unclear.

In this study, we explored the expression of circKIF4A in ovarian cancer tissues. Then, a series of experiments were conducted to investigate how circKIF4A functioned in ovarian cancer *in vitro* and *in vivo*. The results revealed that circKIF4A was highly expressed in ovarian cancer tissues. Knockdown of circKIF4A suppressed cell proliferation and migration in ovarian cancer. Subsequent mechanism study revealed that circKIF4A promoted ovarian cancer progression by sponging miR-127 and upregulated the expression of Junctional adhesion molecule 3 (JAM3). miR-127 has been reported as a tumor-suppressor and a potential diagnostic biomarker for multiple cancers [9–11], while JAM3 has been reported to promote tumor growth and aggressiveness of ovarian cancer [12]. Therefore, circKIF4A could be a novel biomarker and therapeutic target for ovarian cancer.

## RESULTS

### Knockdown of circKIF4A inhibits ovarian cancer proliferation and invasion *in vitro*

**Figure 1:**
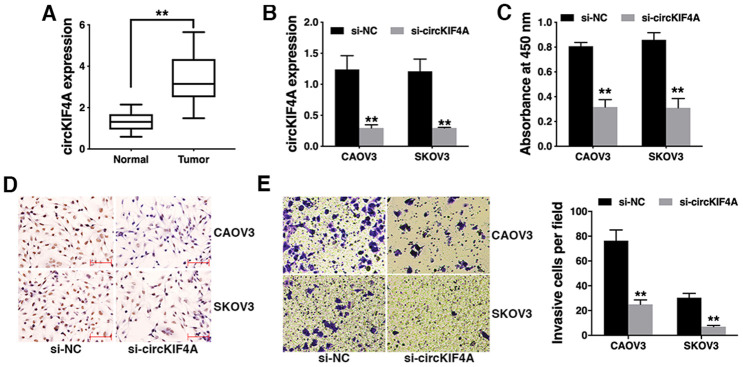
**Knockdown of circKIF4A inhibits ovarian cancer proliferation and invasion *in vitro.*** (**A**) The expression of circKIF4A in ovarian cancer tissues (Tumor) and normal adjacent tissues (Normal). (**B**) circKIF4A was successfully knocked down by siRNA. (**C**) CCK-8 assay was performed to detect cell proliferation. (**D**) BrDU immunostaining of ovarian cancer cells transfected with si-NC of si-circKIF4A. (**E**) Transwell assay was performed to assess cell invasive ability (left) and the number of invasive cells was quantified by ImageJ (right). **P < 0.01

To explore the expression profile of circKIF4A in ovarian cancer, we collected 50 pair of ovarian cancer tissues and adjacent normal tissues and performed qRT-PCR. We found that circKIF4A was significantly upregulated in ovarian cancer tissues (Figure 1A). To explore the function of circKIF4A in ovarian cancer, we used siRNA to knock down the expression of circKIF4A (Figure 1B). CCK-8 assay revealed that circKIF4A knockdown significantly inhibited cell proliferation (Figure 1C). BrDU assay also showed that circKIF4A knockdown reduced proliferation ability of ovarian cancer cells (Figure 1D). Transwell assay showed that cell invasion was significantly suppressed after downregulation of circKIF4A (Figure 1E).

### Knockdown of circKIF4A inhibits ovarian cancer growth and metastasis *in vivo*

**Figure 2:**
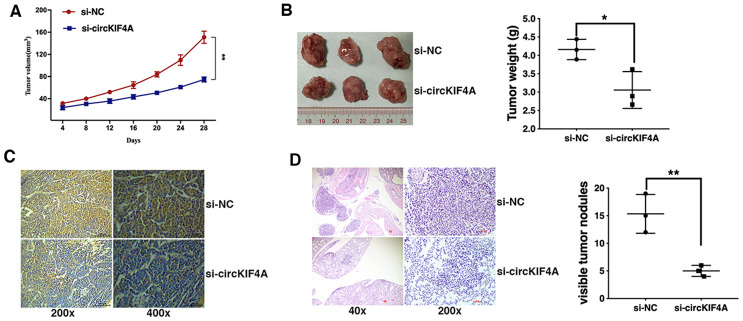
**Knockdown of circKIF4A inhibits ovarian cancer growth and metastasis *in vivo.*** (**A**) The growth curves of tumors were shown. (**B**) Representative images of xenograft tumors (left) and the tumor weight was summarized (right). (**C**) IHC staining of Ki-67 in ovarian cancer cells transfected with si-NC of si-circKIF4A. (**D**) Representative images of lung metastatic nodules on HE-stained sections (left) and the number of metastatic nodules was quantified (right). *P < 0.05, **P < 0.01

To further explore the function of circKIF4A in ovarian cancer *in vivo*, mouse xenograft models were established. circKIF4A inhibition significantly decreased xenograft tumor growth (Figure 2A and 2B) and the expression intensity of Ki-67 in cancer cell (Figure 2C). Lung metastasis was also suppressed by circKIF4A knockdown (Figure 2D), indicating that the knockdown of circKIF4A inhibits ovarian cancer growth and metastasis *in vivo*.

### circKIF4A acts as a sponge for miR-127

**Figure 3:**
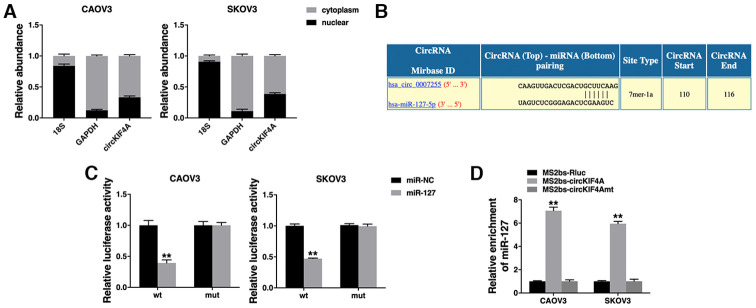
**circKIF4A acts as a sponge for miR-127.** (**A**) The expression levels of nuclear control (18S), cytoplasmic control (GAPDH) and circKIF4A were detected. (**B**) The predicted binding sites of miR-127 within the circKIF4A sequence. (**C**) Luciferase assay of cells co-transfected with miR-127 mimics and wild type or mutant luciferase reporter. (**D**) MS2-based RIP assay transfected with MS2bs-circKIF4A, MS2bs-circKIF4Amt or control. **P < 0.01.

We confirmed the intracellular location of circKIF4A and found it mainly localized in cytoplasm (Figure 3A). Then, we used Circular RNA Interactome (https://circinteractome.nia.nih.gov/index.html) to predict the potential circRNA/miRNA interaction. We found binding sites for miR-127 in circKIF4A sequence (Figure 3B). Luciferase reporter assay revealed that the luciferase intensity decreased after co-transfection of wild type luciferase reporter and miR-127 mimics, while the mutated luciferase reporter had no such effect (Figure 3C). To confirm the direct binding of circKIF4A and miR-127, RIP assay was performed and we found miR-127 predominantly enriched in MS2bs- circKIF4A group (Figure 3D), indicating that circKIF4A directly interacts with miR-127 and could act as a sponge for miR-127.

### circKIF4A acts as a ceRNA to regulate JAM3

**Figure 4:**
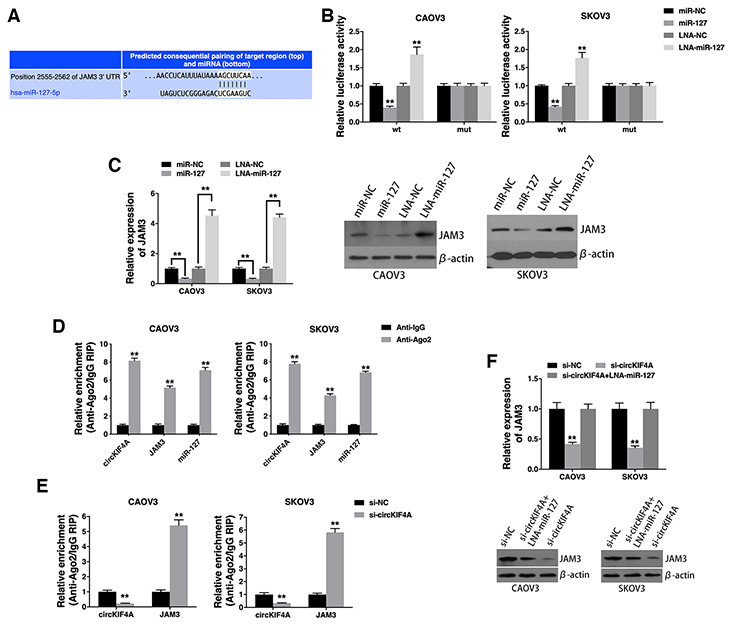
**circKIF4A acts as a ceRNA to regulate JAM3.** (**A**) The predicted binding sites of miR-127 within the JAM3 3’UTR. (**B**) Cells were transfected and luciferase assay was performed. (**C**) Cells were transfected and JAM3 expression was detected by qRT-PCR (left) and western blotting (right). (**D**) RIP assay showing the enrichment of circKIF4A, JAM3 and miR-127 on Ago2. (**E**) Cells were transfected and a RIP assay on Ago2 was performed. (**F**) Cells were transfected and JAM3 expression was detected by qRT-PCR (up) and western blotting (below). **P < 0.01.

To explore whether circKIF4A sponges miR-127 to regulate the expression of its downstream target, we searched TargetScan for target genes of miR-127 and JAM3 was predicted (Figure 4A). Luciferase reporter assay showed decreased luciferase intensity after co-transfection of miR-127 mimics and wild type luciferase reporter (Figure 4B). Moreover, co-transfection of wild type luciferase reporter and miR-127 inhibitor increased luciferase intensity (Figure 4B). And miR-127 suppressed JAM3 expression, while miR-127 inhibitor increased JAM3 expression (Figure 4C), indicating that JAM3 is a downstream target of miR-127 and is regulated by miR-127.

RIP assay on Ago2 further revealed that circKIF4A, JAM3 and miR-127 were mainly enriched to Ago2 (Figure 4D), indicating that circKIF4A and JAM3 are recruited to an Ago2-related RISC where they interact with miR-127. In addition, knockdown of circKIF4A decreased the enrichment of circKIF4A to Ago2, while increasing the enrichment of JAM3 to Ago2 (Figure 4E), indicating that circKIF4A could function as a ceRNA and compete with JAM3 to bind miRNAs. Furthermore, circKIF4A knockdown led to decreased JAM3 expression, while transfection with a miR-127 inhibitor reversed this decrease (Figure 4F), indicating that circKIF4A sponges miR-127 to increase JAM3 expression.

## DISCUSSION

circRNAs are deregulated in various cancers and play vital roles in cancer progression [13]. circRNAs could either act as tumor suppressors or oncogenes in cancer development. In glioma, circFBXW7 inhibited cell proliferation and cell cycle acceleration to repress tumorigenesis [14]. In triple-negative breast cancer, circEPSTI1 promoted cell proliferation and suppressed cell apoptosis [15]. Here in this study, we showed that circKIF4A was highly expressed in ovarian cancer tissues and knockdown of circKIF4A suppressed ovarian cancer cell proliferation, invasion and migration *in vitro* and *in vivo.*

However, the clinical implication of circKIF4A in ovarian cancer need to be clarified. Nowadays, there is still no ideal tumor markers which can be applied into clinical early diagnosis for ovarian cancer [16]. Therefore, new tumor markers with high sensitivity and specificity need to be identified. In the further study, we intend to expand the sample size to explore the relationship between circKIF4A expression and prognosis and clinicopathological features of ovarian cancer to discuss the potential role of circKIF4A as a tumor biomarker in ovarian cancer diagnosis and treatment.

Recently, circRNAs have been reported to function as miRNA sponges [17, 18]. In hepatocellular carcinoma, circMTO1 acts as a sponge for miR-9 to suppress cancer progression [19]. In lung cancer, circTP63 functions as a ceRNA to upregulate FOXM1 expression and promote cancer progression [20]. Here, we showed that circKIF4A could directly interact with miR-127 and act as a sponge for miR-127.

miRNAs are a class of small regulatory RNAs which are associated with tumorigenesis and modulate a variety of biological processes, including cellular differentiation, apoptosis, metabolism, and proliferation. miR-127 has been reported as a tumor-suppressor and can serve as a potential diagnostic biomarker for multiple cancers. In hepatocellular carcinoma, miR-127 is downregulated and overexpression of miR-127 can inhibit cell proliferation and tumorigenicity through downregulating Sept7 expression [9]. miR-127 also decreases the phosphorylation of p65 and the expression of downstream targets of the NF-κB signaling pathway, and inhibits the growth and colony formation of hepatocellular carcinoma cells through decreasing BLVRB expression [21]. Overexpression of miR-127 inhibits hepatocellular carcinoma cell migration, invasion and tumor growth through repressing MMP13 expression and diminishing MMP13/TGFβ-induced cell migration [22]. In gastric cancer, miR-127 is significantly down-regulated and ectopic expression of miR-127 inhibits cell proliferation, cell cycle progression, cell migration and invasion by directly interacting with MAPK4 [10]. In breast cancer, miR-127 is down-regulated and significantly correlated with poorer overall survival [11]. Over-expression of miR-127 inhibits breast cancer cell proliferation, enhances apoptosis, and reduces migration and invasion via targeting BCL6 [23]. However, the role and function of miR-127 in ovarian cancer has not been studied so far. In this study, we found that circKIF4A functioned as a sponge for miR-127 to regulate the expression of JAM3.

JAM3 is the third member of the JAM family, which is a transmembrane protein with significant roles in regulation of cell functions [24]. JAM3 is expressed in most of tumors having potent metastatic properties. The expression of JAM3 promotes metastasis by enhancing both the adhesion of cancer cells to extracellular matrices and the subsequent invasion [25]. JAM3 is highly enriched in leukemia-initiating cells and play an important role in the maintenance of leukemia-initiating cell stemness through LRP5/PDK1/AKT/GSK3β/β-catenin/CCND1 signaling pathways. Knockdown of JAM3 leads to a dramatic decrease in leukemia-initiating cell proliferation [26]. JAM3 promotes cell migration and invasion in melanoma [27] and glioma [28]. In ovarian cancer, JAM3 promotes tumor growth and aggressiveness [12]. Here, we found that circKIF4A sponged miR-127 and up-regulate the expression of JAM3 to promote ovarian cancer progression.

In conclusion, we revealed that circKIF4A was highly expressed in ovarian cancer tissues. Knockdown of circKIF4A suppressed ovarian cancer cell proliferation and migration. circKIF4A up-regulated the expression of JAM3 by sponging miR-127. circKIF4A could be a novel biomarker and therapeutic target for ovarian cancer.

## MATERIALS AND METHODS

### Ethical standards

This study was approved by the Ethics Committee of The First Affiliated Hospital of University of South China and conducted according to the Helsinki Declaration. Informed consents were obtained from all patients. Animal study was approved and conducted according to the Institutional Animal Care and Use Committee (IACUC) of The First Affiliated Hospital of University of South China.

### Clinical samples

50 pair of ovarian cancer tissues (Tumor) who were diagnosed by histopathology and did not receive any chemotherapy and radiation therapy before, and adjacent normal tissues (Normal) were collected from The First Affiliated Hospital of University of South China and immediately stored in RNAlater (Ambion, USA) for qRT-PCR analysis.

### Cell culture and transfection

Ovarian cancer cell lines CAOV3 and SKOV3 were obtained from American Type Culture Collection (USA) and cultured in RPMI-1640 medium supplemented with 10% fetal bovine serum. Cell lines were confirmed free of mycoplasma infection and cell authenticity was verified by DNA fingerprinting.

Cells were transfected with Lipofectamine 2000 (Invitrogen, USA). siRNAs targeting circKIF4A were synthesized by GenePharma (China), the sequences are presented in Supplementary Table 1. miR-127 mimic and inhibitor were purchased from GeneCopoeia (USA).

### Quantitative real-time PCR (qRT-PCR)

Total RNA was isolated by TRIzol (Invitrogen) and the nuclear and cytoplasmic fractions were isolated by NE-PER™ Nuclear and Cytoplasmic Extraction Reagents (Thermo Scientific). qRT-PCR was conducted with SYBR Premix Ex Taq™ (Takara, Japan) and an All-in-One™ miRNA qRT-PCR Detection Kit (GeneCopoeia) using Bio-Rad IQTM5 Multicolor Real-Time PCR Detection System (USA). The primers for qRT-PCR were purchased from Invitrogen (Supplementary Table 1).

### Cell counting kit-8 (CCK-8) assay

Cells (1 × 10^3^) were seeded and CCK-8 solution (Dojindo Laboratories, Japan) was added. After 2h of incubation at 37°C, the absorbance at 450nM was measured by microtiter plate reader (Bio-Tek EPOCH2, USA).

### Bromodeoxyuridine (BrDU) assay

Cell proliferation was also determined by measuring incorporation of the thymidine analogue BrDU into host DNA. Cells (1×10^4^) were seeded and exposed to an acute pulse of BrDU (10mg/ml) for 2h. Cells were then fixed with cold 4% paraformaldehyde and visualized for the presence of BrDU incorporation by immunocytochemistry, and photographed using standard light microscopy.

### Transwell assay

Transwell assays were performed with migration chambers (BD Biosciences, USA). Briefly, cells (1×10^4^) were seeded and medium with 10% FBS was added to the lower chamber as a chemoattractant. After 24 h, cells were fixed in methanol, stained with 0.1% crystal violet and counted.

### Mouse xenograft model

Cells (2×10^6^) were subcutaneously injected into the dorsal flanks of 4-week-old female BALB/c nude mice (three mice per group to provide a power of 90% for a significance level of 0.05 with a two-tailed t test). Then the mice were intratumorally injected with 40 μL si-NC or si-circKIF4A every 4 days. Xenografts were excised under anesthesia after 4 weeks, and the tumor weights were measured.

For lung metastasis studies, cells (1 × 10^5^) were injected into the mice tail veins (three mice per group to provide a power of 90% for a significance level of 0.05 with a two-tailed t test). Then the mice were injected with 40 μL si-NC or si-circKIF4A every 4 days. After 8 weeks, the lungs were excised under anesthesia, and the numbers of macroscopically visible lung metastatic nodules were counted and validated by assessment of hematoxylin and eosin (HE)-stained sections by microscopy.

### Immunohistochemistry (IHC) analysis

After deparaffinizing and rehydrating, the slides were treated with 90% methanol/3% H_2_O_2_ solution for 10 minutes at room temperature to block endogenous peroxidase. Then, the slides were soaked in sodium citrate buffer (10 mM Sodium citrate, 0.05% Tween 20, pH 6.0) under 96°C for 5 min for antigen retrieval. After blocking by BSA, antibody against Ki-67 (dilution 1:400, CST, USA) was used and incubated overnight at 4°C then incubated at room temperature with biotinylated secondary antibody for 10 minutes, and finally HRP-Streptavidin for 10 minutes. After DAB staining, the intensity of Ki-67 staining was imaged.

### Luciferase reporter assay

The circKIF4A sequences including the miR-127 binding sites was inserted into the pGL3 luciferase vector (Promega, USA) immediately downstream of luciferase. Mutations in the miR-127 seed-region were conducted with Fast Site-Directed Mutagenesis Kit (TIANGEN, China). The JAM3 3’-UTR including the miR-127 binding sites was inserted into the pGL3 luciferase vector. Mutations in the miR-127 seed-region served as a mutant control.

Cells (5×10^3^) were seeded and cotransfected with corresponding vectors and miR-127 mimic or miR-127 inhibitor. After 48 h of incubation, luciferase intensity was measured by dual-luciferase reporter assay system (Promega).

### RNA immunoprecipitation (RIP) assay

Cells were co-transfected with MS2bs-circKIF4A, MS2bs-circKIF4Amt or MS2bs-Rluc and MS2bp-GFP. After 48 h, RIP was performed with Magna RIP RNA-Binding Protein Immunoprecipitation Kit (Millipore, USA). The RNA complexes were then purified, and the level of miR-127 was quantified.

For the RIP assay for Ago2, RIP was performed with an anti-Ago2 antibody (Millipore). RNAs were then purified, and the levels of circKIF4A, JAM3 and miR-127 were measured.

### Statistical analysis

Statistical analysis was conducted with SPSS 19.0 software. Comparisons between groups were performed using t tests. Unless otherwise indicated, data are presented as the mean ± S.D. of three independent experiments. *P* < 0.05 was considered statistically significant.

## Supplementary Material

Supplementary Table 1
