# Supplementary material for: circKIF4A sponges miR-127 to promote ovarian cancer progression
Source: Aging (Albany NY). 2020 Aug 5;12(18):17921–9. doi: 10.18632/aging.103389 (PMC7585112; doi:10.18632/aging.103389)
Supplement: Supplementary Table 1 [file aging-12-103389-s001..pdf]

SUPPLEMENTARY TABLE

Supplementary Table 1. The sequences of siRNAs and primer sequences for qRT-PCR used in this study.

The sequences of siRNAs used in this study

siRNA	Species	Sequences
si-NC	Human	UUCUCCGAACGUGUCACGUTT
si-circKIF4A	Human	GCCUGGAUCUAUAACGUAUTT

Primer sequences for qRT-PCR used in this study

Construct	Species	Direction	Sequence (5' - 3')
circKIF4A	Human	Forward	GAGGTACCCTGCCTGGATCT
		Reverse	TGGAATCTCTGTAGGGCACA
18S	Human	Forward	TTAATTCCGATAACGAACGAGA
		Reverse	CGCTGAGCCAGTCAGTGTAG
β-actin	Human	Forward	AGCGAGCATCCCCCAAAGTT
		Reverse	GGGCACGAAGGCTCATCATT
GAPDH	Human	Forward	GGAGCGAGATCCCTCCAAAAT
		Reverse	GGCTGTTGTCATACTTCTCATGG
JAM3	Human	Forward	TCCAGCAATCGAACCCCAG
		Reverse	CTTGTCTGCGAATCCGTAATGAT
